# Multi-Omics Profiling Identifies Risk Hypoxia-Related Signatures for Ovarian Cancer Prognosis

**DOI:** 10.3389/fimmu.2021.645839

**Published:** 2021-07-19

**Authors:** Xingyu Chen, Hua Lan, Dong He, Runshi Xu, Yao Zhang, Yaxin Cheng, Haotian Chen, Songshu Xiao, Ke Cao

**Affiliations:** ^1^ Department of Oncology, Third Xiangya Hospital of Central South University, Changsha, China; ^2^ Department of Obstetrics and Gynecology, Third Xiangya Hospital of Central South University, Changsha, China; ^3^ Department of Respiration, The Second People’s Hospital of Hunan Province of Hunan University of Chinese Medicine, Changsha, China; ^4^ Medical school, Hunan University of Chinese Medicine, Changsha, China

**Keywords:** hypoxia, tumor immune microenvironment, immune-escape, immune response, somatic copy number alterations

## Abstract

**Background:**

Ovarian cancer (OC) has the highest mortality rate among gynecologic malignancy. Hypoxia is a driver of the malignant progression in OC, which results in poor prognosis. We herein aimed to develop a validated model that was based on the hypoxia genes to systematically evaluate its prognosis in tumor immune microenvironment (TIM).

**Results:**

We identified 395 hypoxia-immune genes using weighted gene co-expression network analysis (WGCNA). We then established a nine hypoxia-related genes risk model using least absolute shrinkage and selection operator (LASSO) Cox regression, which efficiently distinguished high-risk patients from low-risk ones. We found that high-risk patients were significantly related to poor prognosis. The high-risk group showed unique immunosuppressive microenvironment, lower antigen presentation, and higher levels of inhibitory cytokines. There were also significant differences in somatic copy number alterations (SCNAs) and mutations between the high- and low-risk groups, indicating immune escape in the high-risk group. Tumor immune dysfunction and exclusion (TIDE) and SubMap algorithms showed that low-risk patients are significantly responsive to programmed cell death protein-1 (PD-1) inhibitors.

**Conclusions:**

In this study, we highlighted the clinical significance of hypoxia in OC and established a hypoxia-related model for predicting prognosis and providing potential immunotherapy strategies.

## Introduction

Ovarian cancer (OC) has the highest mortality rate among gynecologic malignancies, with an estimated 384,000 deaths in 2018 worldwide (1). Despite recent advances in OC treatment, most OC patients diagnosed at advanced stages and have poor prognoses, with a recurrence rate of 70% within 3 years and only 30% 5-year survival rate (2, 3). Immunotherapy is a promising treatment strategy for many cancers, and immunotherapy has led to improved quality of life and prolonged survival for some OC patients (4–6). However, immunotherapy for ovarian cancer still faces challenges, with only 8-9% objective response and a lack of reliable biomarkers to predict response (7). Therefore, there is an urgent need to identify sufficient and reliable biomarkers with high specificity and sensitivity for OC patients to distinguish responsive patients are suited to immune checkpoint inhibitor therapy from OC patients.

The hypoxic microenvironment plays a key role in tumorigenesis, radiotherapy, and chemotherapy resistance in OC (8, 9). Particularly, hypoxia affects the tumor microenvironment and promotes tumor angiogenesis, the release of damage-associated pattern molecules, tumor immunosuppression, and immune escape (10, 11). Hypoxia is also vital in the natural anti-tumor immune response as it can reduce the activity of NK or CTL cells (12). At the same time, hypoxia modulates inhibitory cells, including tumor-associated macrophages (TAMs) and neutrophils, and increases the levels of immunosuppressive molecules, including TGFB, IL4, and IL10, which result in immune suppression and evasion (13, 14). In recent years, immunosuppressive blockers, such as the inhibitors of programmed death ligand 1 (PD-L1) or the cytotoxic T-lymphocyte-associated protein-4 (CTLA-4), have shown potential as novel treatment targets (15, 16); however, only some patients may benefit from immunotherapy (17). Therefore, it is of great significance to explore the common mechanisms in hypoxia, immune status, and OC microenvironment.

Whole-genome sequencing provides unlimited opportunities to systemically explore the tumor microenvironment. However, due to inadequate sample size and the lack of available multi-omics data, only a few studies have applied genomic analysis to study OC from an immunological perspective. In our previous study, based on multi-omics data from the Cancer Genome Atlas (TCGA) cohort, we found that autophagy can affect the OC immune microenvironment (18). In the present study, to further explore the immune microenvironment of OC, we studied the impact of hypoxia on the immune microenvironment of OC. We identified hypoxia-related genes using weighted gene co-expression network analysis (WGCNA), established a risk model, and verified prognostic signatures. Then, we evaluated the immune infiltration landscape and proposed potential tumor escape mechanisms in high- and low-risk patients. In addition, we used TIDE and SubMap algorithms to predict OC immunotherapy responsiveness. Finally, a nomogram-based risk assessment and clinicopathological features of patients were constructed to improve the prediction ability and accuracy. The risk signature we obtained may help provide new prognostic biomarkers for effective immunotherapy in OC and identify patients’ responsiveness to immunotherapy, improve the individualized prognosis of OC patients, and assist clinicians in making optimal treatment decisions.

## Materials and Methods

### Ovarian Cancer Dataset and Preprocessing

Gene expression data, patient clinical information, somatic mutation status data, and the waterfall diagram of significant tumor mutational burden (TMB) (generated by the maftools package in R) in OC were downloaded from TCGAwebsite (https://portal.gdc.cancer.gov/repository). There were 587 patients with OC in the TCGA-OV data, all patients in TCGA-OV were diagnosed as serous cystadenocarcinoma. Among the 520 patients with sequencing data from of tissue samples, two patients had no prognostic data and one patient was duplicated. Therefore, 517 patients were included in this study. GISTIC_2.0 was used for copy number analysis to identify amplified or deleted genomes (19). The burden of copy number alterations was calculated as the total number of genes whose copy number changes at the lesion and arm levels. Hypoxia gene was obtained from GeneCards (https://www.genecards.org/). MRNAs with a relevance score of ≥1 were selected and expressed in the TCGA database, with a total of 1776 genes identified.

### Weighted Gene Co-Expression Network Construction and Module Identification

WGCNA is a systems biology algorithm based on high-throughput gene expression profiling. WGCNA searches gene modules with cooperative expression and identifies correlations between modules and phenotypes (20). In this study, WGCNA was performed using the WGCNA R package. Soft threshold power β = 4, scale-free R^2^ = 0.97, and the pickSoftThreshold function were used to construct a standard scale-free network. The correlation between the modules and immune cells was evaluated using Pearson’s correlation coefficient analysis. Two modules with the highest average gene significance scores among all genes in the modules were selected as candidate modules related to immune infiltration.

### Estimation of Immune Infiltration

CIBERSORT (https://cibersort.stanford.edu/), a deconvolution algorithm based on the expression profile of 547 genes, was performed to accurately determine the absolute abundance of 22 immune cell populations, i.e., memory B cells, plasma cells, naive B cells, follicular helper T cells, CD8 T cells, naïve CD4 T cells, resting memory CD4 T cells, macrophages M_2_, activated memory CD4 T cells, monocytes, T cells regulatory (Tregs), resting NK cells, gamma delta T cells, macrophages M_0_, activated NK cells, macrophages M_1_, resting dendritic and mast cells, activated dendritic cells, eosinophils, activated mast cells, and neutrophils (21). In addition, the Tumor Immune Estimation Resource (TIMER, https://cistrome.shinyapps.io/timer/) database was used to calculate the copy number of hypoxia risk signatures and the infiltration level of immune cells, including CD8^+^ T-cells, dendritic cells, macrophages, B-cells, CD4^+^ T-cells, and neutrophils (22, 23).

### Construction and Verification of the Prognostic Model

To screen the best risk genes, 517 samples were randomly divided into training or validation sets (6:4) to identify and evaluate the predictors. Univariate Cox regression analysis was performed to select the significant genes associated with survival among 395 hypoxia-related genes in the red and blue modules. In addition, LASSO regression analysis was conducted to screen optimal gene combination for identifying prognostic risk signatures. Cox regression analysis was performed to further identify the selected genes. The hypoxia-related risk score formula was calculated as follows (24, 25):

Risk score=Expression mRNA1×Coefficient mRNA1×Expression mRNA2 ×Coefficient mRNA2×…Expression mRNAn.

OC patients were classified into high- or low-risk groups according to the “surv-cutpoint” function. In 181 high-risk cases, there were 16 cases of preoperative and 19 cases of postoperative adjuvant therapy. There were 336 patients in the low-risk group, 60 patients underwent preoperative adjuvant therapy, while 27 patients underwent postoperative adjuvant therapy. Survival analysis was performed using the Kaplan–Meier curve. The area under the curve (AUC) of the time-dependent receiver operating characteristic (tROC) curves (“timeROC” package in R) was set as the indicator of prognostic efficacy. Subsequently, univariate and multivariate Cox regression analyses were used to analyze the relationship between risk score and patient clinical features (i.e., age, tumor grade, stage, lymph node metastasis, and survival status) using the survminer package in R. The prognostic value of each hypoxia-related gene was also assessed. Hazard ratios (HRs) and the corresponding 95% confidence intervals (CIs) were calculated.

### Functional and Pathway Enrichment Analysis

Limma R package was used to identify the signaling pathways that were differentially activated between the low- and high-risk groups (26). Relative genes were analyzed using gene ontology (GO) and Kyoto Encyclopedia of Genes and Genomes (KEGG) with the corrected *p* < 0.05 to determine the significance of the genes. Gene ontology (GO) database includes biological process (BP), molecular function (MF), and cellular component (CC). KEGG identifies significantly enriched mRNA biological pathways. To identify the up-regulated and down-regulated signal pathways specific for the tumor microenvironment (TME) phenotype between the high- and low-risk groups, Gene Set Enrichment Analysis (GSEA) was conducted with adjusted *p* < 0.05 using the cluster filer R package (27).

### Development of Prognostic Nomogram Based on Hypoxia-Related Signatures

We used clinical risk factors and the multivariate Cox regression coefficients of the risk score based on the hypoxia-related signatures to construct a nomogram. The prognostic nomogram was established with the “rms” package in R (28). The prediction accuracy of the nomogram was evaluated using the consistency index (C-index) and a calibration curve (29).

### Prediction of Immunotherapy Response

Immune checkpoint blockades that target CTLA-4 and PD-1/PD-L1 have shown some promise against malignancies (30, 31). Tumor immune dysfunction and exclusion (TIDE) and SubMap algorithm were used to predict the clinical response to immune checkpoint inhibitors. TIDE is a calculation method that uses gene expression profiles to predict ICB response. It evaluates two different tumor immune escape mechanisms, including the dysfunction of tumor-infiltrating cytotoxic T lymphocytes (CTL) and the rejection of CTL by immunosuppressive factors (32). SubMap was used to compare the similarity of expression profiles; this feature can be reflected as a treatment response (33). We used the SubMap algorithm to predict the possibility of anti-PD1 and anti-CTLA4 immunotherapy response. The expression profile of the high- and low-risk group that we defined with a published dataset containing 47 patients with melanoma and related annotation data was obtained from the **Supplementary Materials** of Lu X et al (34).

### Statistical Analysis

All statistical calculations were performed using R (v3.6.1, http://www.R-project.org). The correlation matrices were conducted using Pearson or Spearman correlation. The Wilcoxon test and Kruskal–Wallis test were performed to compare continuous variables and ordered categorical variables, respectively. The false discovery rate (FDR) correction was used to adjust the *P* value for multiple tests. All tests were two-sided, and *p* < 0.05 was considered statistically significant.

## Results

### Study Design

**Figure 1:**
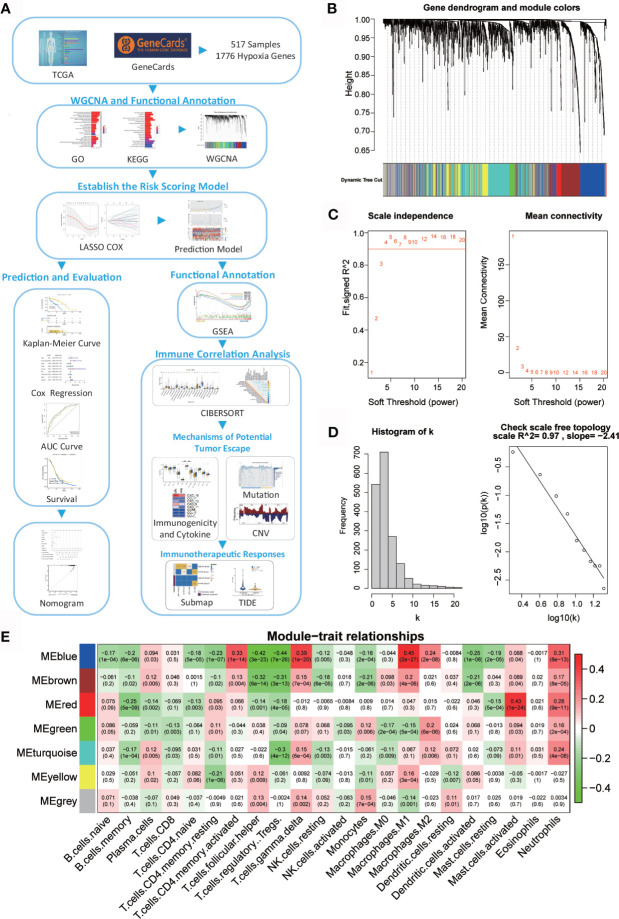
Network of co-expressed genes and module–trait relationships. **(A)** Flow diagram of this study’s systematic analysis and validation. **(B)** Dendrogram of the differentially expressed genes clustered based on different metrics. Each branch in the figure represents one gene; each color indicates a single module that contains weighted co-expressed genes. **(C)** The left panel presents the relationship between the soft-threshold and scale-free R2. The right panel presents the relationship between the soft-threshold and mean connectivity. **(D)** Verification of the scale-free network. **(E)** Heatmap of the correlation between the module eigengenes and the immune cells in ovarian cancer. Each column contains the corresponding correlation and p value.

A flow chart was designed to systematically describe the study design (**Figure 1A**). Hypoxia genes were downloaded from GeneCards, and 1776 mRNAs were selected with a relevance score ≥ 1 and expressed in the TCTA database. GO and KEGG enrichment analysis were then performed on 1776 genes. WGCNA showed that two modules are highly associated with the immune system. GSEA was performed for functional annotation. Next, a hypoxia-related risk signature by LASSO Cox regression was established, which validated the reliability of the risk signature with the tROC curve, Kaplan-Meier curve, and Cox regression. Then, we analyzed the difference in immune infiltration, mutations, copy number variation, and response to immunotherapy between high- and low-risk patients. Finally, a nomogram was developed based on the risk signature and clinicopathological factors. A calibration plot was constructed to predict the 1-, 3-, and 5-year survival rates of patients with OC.

### Weighted Co-Expression Network Construction and Key Module Identification

A total of 517 TCGA-OV samples with data were included, of which 1776 genes were received for WGCNA analysis (**Supplementary Figure 1A**). WGCNA analysis identified six modules (**Figure 1B**; non-clustered hypoxia-related genes are shown in gray). Through the definition of module connectivity (**Figures 1C, D**) and the absolute value of the Pearson correlation and immune cell relationship (**Figure 1E**), it was found that the red and blue modules showed a high correlation with immune cells (**Supplementary Figures 1B–F**), these two modules were used for further analysis. The blue module was significantly related to macrophage M_1_, T cells gamma delta, and T cells CD4 memory activated, and the red module was highly associated with mast cells activated.

To reveal the potential biological functions of the genes in the blue and red module, we conducted GO and KEGG analyses (**Supplementary Figures 2A, B**). The genes in these two modules are closely related to immunity. The enriched GO terms were a response to decreased oxygen levels, hypoxia, and cytokine activity. KEGG pathway analysis revealed that these modular genes were related to cytokine–cytokine receptor interaction, apoptosis, PI3K-Akt and MAPK signaling pathways, cytokine–cytokine receptor interaction, and TNF signaling pathway.

### Establishment and Verification of the Hypoxia-Immune Related Prognostic Signature

**Figure 2:**
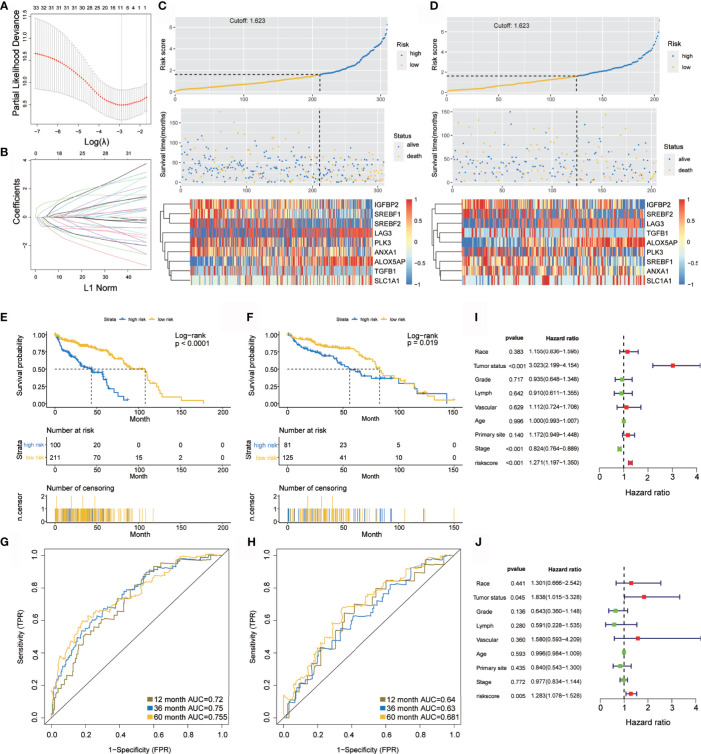
Construction and verification of the hypoxia prognostic classifier. **(A, B)** Determination of the number of factors using LASSO analysis. **(C, D)** Risk score distribution, survival overview, and heatmap in patients in the TCGA training cohort **(C)** and the TCGA internal validation cohort **(D)** datasets assigned to high- and low-risk groups based on the risk score. **(E, F)** Kaplan-Meier curve for the TCGA training cohort **(E)** and the TCGA internal validation cohort **(F)**. **(G, H)** ROC curve of the TCGA training cohort **(G)** and the TCGA internal validation cohort **(H)**. **(I, J)** Univariate **(I)** and multivariate **(J)** Cox regression analysis of risk score, age, tumor status(with tumor or tumor free), grade, lymphovascular invasion, vascular invasion, primary site and stage. ROC, receiver operator characteristic. AUC, the area under the curve.

The prognostic model showed a powerful predictive function (35, 36). LASSO-COX analysis identified nine hypoxia-related genes (IGFBP2, SREBF2, LAG3, TGFB1, ALOX5AP, PLK3, SREBF1, ANXA1, and SLC1A1) that were included in the risk score (**Figures 2A, B**). Since hypoxia usually promotes a more aggressive tumor phenotype (37), the prognostic value of the risk score was further investigated. According to the optimal cut-off value, OC patients were divided into high- or low-risk groups. It was shown that the mortality rate of the high-risk group in the TCGA-OV training and internal validation cohorts was significantly higher than that of the low-risk group (**Figures 2C, D**). To compare the sensitivity and specificity of the risk score to the prognostic value of OC, Kaplan–Meier curves were plotted to analyze the survival of the hypoxia-immune gene signature.

Kaplan–Meier analysis demonstrated that the high-risk group predicted poorer overall survival than that predicted for the low-risk group (**Figure 2E**), which was further verified by the TCGA internal validation set (**Figure 2F**). The ROC curve showed a high significance for survival in OC, and was studied as a continuous variable. The AUC was 0.72 at 1 year, 0.75 at 3 years, and 0.755 at 5 years (**Figure 2G**); this was further validated by a validation cohort (**Figure 2H**). These results are encouraging and highlight the high reliability of the predictive value.

Univariate and multivariate Cox regression analyses were applied to assess the independent prognostic value of the hypoxia-immune risk signature in terms of overall survival (OS) of OC patients. Among various clinicopathological variables, univariate analysis emerged as a significant predictor of poor OS (HR = 1.271 [1.197–1.35], *p* < 0.001) (**Figure 2I**). Subsequent multivariate analysis revealed that the risk score was an independent factor for predicting poor OS in patients with OC (HR = 1.283 [1.078–1.528], *p* = 0.005) (**Figure 2J**). Taken together, our results confirmed that hypoxia-immune risk signature was considerably robust, may be better than the currently used clinicopathologic features, and serves as an independent predictor of survival in OC.

### Prognostic Value of Hypoxia-Related Signatures and the Correlation With Tumor-Infiltrating Immune Cells in OC

**Figure 3:**
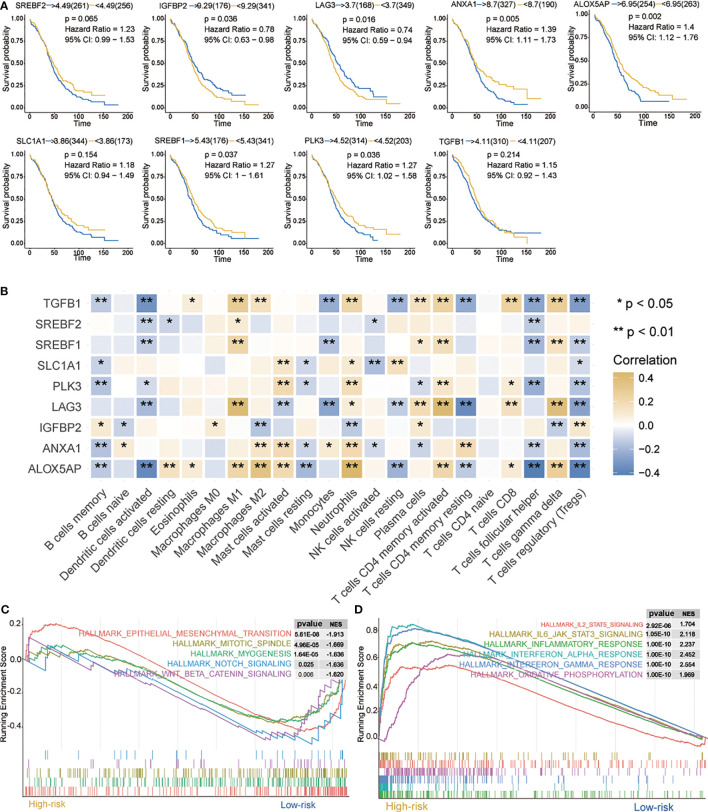
Characteristics of 9 hypoxia signatures. **(A)** Prognosis of 9 hypoxia signatures. **(B)** Correlations between 9 hypoxia signatures and the distribution of immune cell infiltration. **(C, D)** GSEA enrichment in high- and low-risk groups. Normalized enrichment score (NES) > 1 and nominal p-value (NOM p-val) < 0.05 were considered significant. *P < 0.05, **P < 0.01.

Current data suggest that hypoxia may play an important role in immune response and actively interact with immune cells (38). First, prognosis of the nine signatures (**Figure 3A**)—*ALOX5AP*, *ANXA1*, *IGFBP2*, *PLK3*, *LAG3*, and *SREBF1*—showed statistical significance (*p* < 0.05) in OC. Indeed, higher *ALOX5AP*, *ANXA1*, *PLK3*, and *SREBF1* mRNA levels were significantly associated with shorter OS (*p* < 0.05), indicating that these four signatures are risk factors (HR > 1). Of particular note, *LAG3* and *IGFBP2* have lower mRNA levels and better prognosis, suggesting that these two genes may be protective factors (HR < 1).

We further evaluated the correlation between nine hypoxia risk signatures and 22 immune cells. As shown in **Figure 3B**, nine signatures mostly showed a positive correlation with “B cells memory,” “Dendritic cells activated,” “Monocytes,” “Mast cells resting,” “NK cells activated,” “T cells follicular helper,” and “Tregs”, and a negative correlation with “Macrophages M_0_,” “Macrophages M_1_,” “Macrophages M_2_,” “Mast cells activated,” “Neutrophils,” “T cells CD4 memory activated,” “T cells CD8” and “T cells gamma delta.” Among them, *ALOX5AP*, *TGFB1*, *LAG3*, and *IGFBP2* were positively or negatively correlated with most immune cells, which suggested that hypoxia-related signatures had pivotal regulatory effect on the TIM for OC patients.

Next, we analyzed biological processes associated with the differentially expressed genes (DEGs) on red and blue modules. DEGs were identified as shown in **Supplementary Figure 4A**. GO and KEGG enrichment analysis were then provided for the annotation of candidate genes (**Supplementary Figure 4B, C**). GSEA was performed when comparing the high- and low-risk groups. It was observed that IL2/STAT5 signaling, IL6/JAK/STAT3 signaling, interferon response, interferon alpha response, interferon alpha gamma response, and oxidative phosphorylation were up-regulated in the high-risk group (**Figure 3C**). In contrast, in the low-risk group, mesenchymal transition, mitotic spindle, myogenesis, notch signaling, and WNT/β-catenin signaling were downregulated (**Figure 3D**). All these pathways are related to immunity and may cause biological pathways or functional imbalances in the high- and low-risk groups. Our analysis offers useful data on the study of OC pathogenesis and clinical treatment strategy.

### Immune Landscape Between Low- and High-Risk Groups

**Figure 4:**
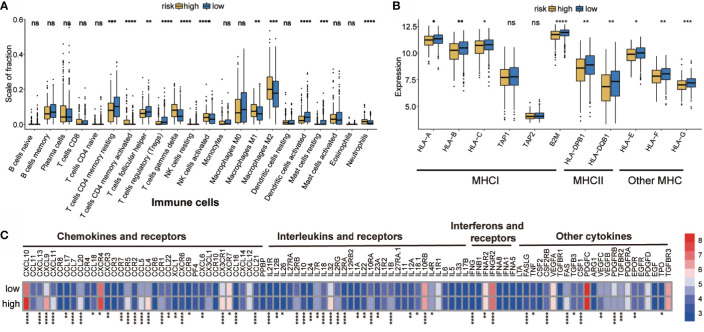
Potential intrinsic immune escape mechanisms of ovarian cancer. **(A)** Abundance of each TME infiltrating cell in high- and low-risk groups. **(B)** Expression of MHC in high- and low-risk groups. **(C)** Differential mRNA expression of chemokines, ILs, IFNs, and other important cytokines and their receptors in high- and low-risk groups (categorized by the median value). The upper and lower ends of the boxes represent the interquartile range of values. The lines in the boxes represent the median value, and the black dots show the outliers. *p < 0.05, **p < 0.01, ***p < 0.001, ns, *p < *0.05, ****P < 0.0001.

Immune landscape was estimated between the high- and low-risk groups. As shown in **Figure 4A**, most of the 22 tumor immune cell types showed significant differences between the high-risk and low-risk groups. In the high-risk group, T cells CD4 memory active, T cells gamma delta, NK cells activated, Macrophages M_1_, Macrophages M_2_, and neutrophils had higher infiltration levels. However, in T cell CD4 memory resting, T cells follicular helper, Tregs, dendritic cells activated, and mast cell activation, were significantly lower in the high-risk group than those in the low-risk group. In addition, the proportion of 22 immune cells was weak- to moderately-correlated (**Supplementary Figure 3**). Differences between groups illustrated that the variations in proportions of tumor-immune cells may be associated with the OS of OC patients. Therefore, immune infiltration heterogeneity in OC may serve as an effective prognostic indicator, which has crucial and practical clinical significance.

### Correlation of the High- and Low-Risk Groups With Tumor Immunogenicity and Cytokine

Hypoxia suppressed tumor immunogenicity by adjusting tumor cells and the immune microenvironment. Significant differences in tumor immune infiltration between the high- and low-risk groups were found. Therefore, whether the groups have a unique immune escape mechanism remains to be studied.

Tumor immunogenicity can directly mediate tumor immune evasion (39). Low antigen presentation ability leads to low immunogenicity, which interferes with the anti-tumor function of tumor cells and adaptive immunity (40). The tumor antigen presentation ability was then analyzed and it was found that the high-risk hypoxia group showed lower expression of MHC I, MHCII, and other MHC-related antigen-presenting molecules (**Figure 4B**), indicating that the high-risk group has antigen presentation defects, thus resulting in immune escape.

Evidence suggests that the inability to attract innate immune cells, inactivation of innate immune chemotaxis, and increase of immunosuppressive molecules after immune stimulation may promote tumor external immunity (41). Moreover, microenvironmental components, other than tumor cells, also contribute to immune escape (42–44). It was found that the high-risk group had higher expression of immunosuppressive cells (neutrophils). It was interesting to observe that both M_1_ and M_2_ macrophages were more abundant in the high-risk group; therefore, a ratio of M_1_/M_2_ may be more informative when evaluating the tumor microenvironment (45). The expression of activated innate immune cells (dendritic cells) in the high-risk group was significantly reduced. In the high-risk group, the immunosuppressive factor levels were higher than those in the low-risk group, such as of chemokines and receptors (e.g., CCR5, CCR7, CCR9, CXCL10, CXCL9, and CXC4), interferons and receptors (e.g., IFNB1, IFNAR2, and IFNGR2), and interleukins and receptors (e.g., IL10RA, IL10RB, IL12, IL32, IL4R) (**Figure 4C**). The high-risk group showed obvious immunosuppression, which may have prevented immune cells from clearing the tumor, but may have also resulted in tumor cells evading immune surveillance and cell death (46).

### Genomic Features in High- and Low-Risk Groups

Genome abnormalities, such as somatic copy number alterations (SCNAs) and mutations, play an important role in tumor immune escape. Therefore, we explored the difference between the high- and low-risk groups at the genomic level.

Somatic mutation is an important cause of tumorigenesis (47, 48). Hence, the TMB of TCGA-OV patients in low- and high-risk groups based on somatic mutation data was analyzed. To elucidate the underlying genomic mechanism, we studied the top 20 mutated genes. The most significant types of mutation were as follows: missense mutations, frame insertions or deletions, nonsense mutations, and distribution of shear sites (**Supplementary Figure 5A**). There were differences in the mutations between different genes. *TP53*, *TTN*, and *MUC16* were the most commonly mutated genes in the cohort, and the mutations occurred in 76%, 33%, and 12% of the cases, respectively. Further analysis revealed that missense mutations were the most common and that single-nucleotide polymorphisms were more common than that was deletion or insertion (**Supplementary Figure 5B**). In OC, C > T occurred most frequently among other single‐nucleotide variants. In addition, the number of mutant genes in each patient was counted, and the mutation categories are presented as a box plot with different colors (**Supplementary Figure 5C**).

**Figure 5:**
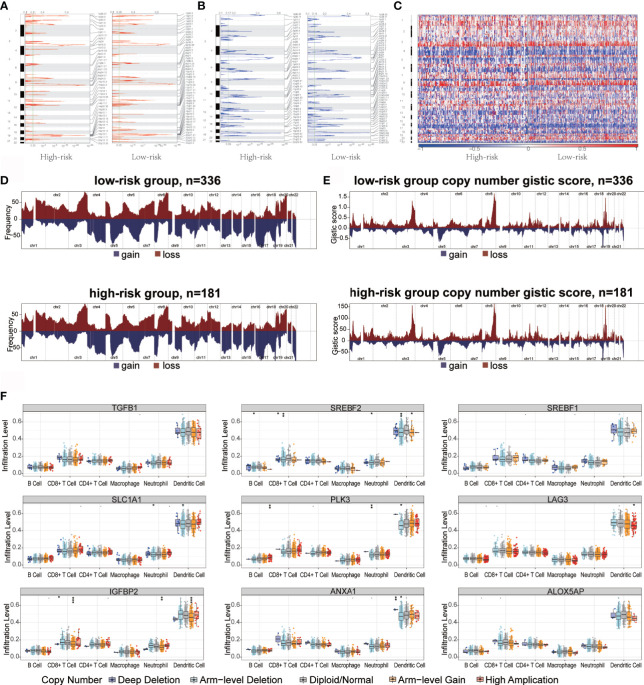
Copy number variation in high- and low-risk groups immune infiltration in ovarian cancer. **(A, B)** Copy number profiles for high- and low-risk groups, with gains in dark red and losses in midnight blue. Gene segments are placed according to their location on chromosomes, ranging from chromosome 1 to chromosome 22. **(C)** Heat map of the differences in copy numbers in 22 chromosome between the high- and low-risk groups. **(D, E)** The left plot **(D)** illustrates the frequency of the gains and losses. The right plot **(E)** shows the cytoband with focal amplification and focal deletion generated using the GISTIC_2.0 software. The q value of each locus is plotted horizontally. **(F)** Effect of genetic alterations on hypoxic signatures of immune cell infiltration. *P < 0.05, **P < 0.01, ***P < 0.001.

Recent studies have shown that the high burden of copy number loss is positively correlated with anti-PD-1 and anti-CTLA-4 blockade resistance, indicating that copy number loss is closely related to tumor immunity (34). CNA was identified between high- and low-risk groups, showing that genes exhibit significant amplification or deletion (**Supplementary Table 1**). Both high- and low-risk groups exhibited genomic amplifications and deletions (**Figures 5A–C**); particularly the gain in chromosome arms 3q, 8q, and 19p and the loss of chromosomes 1p, 5q, and 18q. **Figures 5D, E** show the distribution of the G-score across all chromosomes in different groups. The effects of CNAs of the hypoxia-related signatures on immune infiltration were further analyzed to evaluate the mechanisms by which the risk score was correlated with different immune cell infiltrations. The CNAs of the identified hypoxia-related gene signatures, containing arm-level deletion and gain, markedly influenced the infiltration levels of CD8^+^ T cells, neutrophils, CD4^+^ T cells, macrophages, B cells, and dendritic cells in OC patients (**Figure 5F**). Overall, our analysis indicated that certain genomic changes may lead to decreased immune penetration, thereby affecting immunotherapy.

These results demonstrated that tumor antigen presentation defects, recruitment of inhibitory immune cells and immunosuppressive factors, and change in tumor microenvironment results in the evasion of the monitoring, recognition, and attack by the immune system in high-risk patients, thus promoting tumor escape. In addition, genomic variation may also be involved in immune escape in high-risk groups.

### Immunotherapeutic Response of High- and Low-Risk Patients With OC

**Figure 6:**
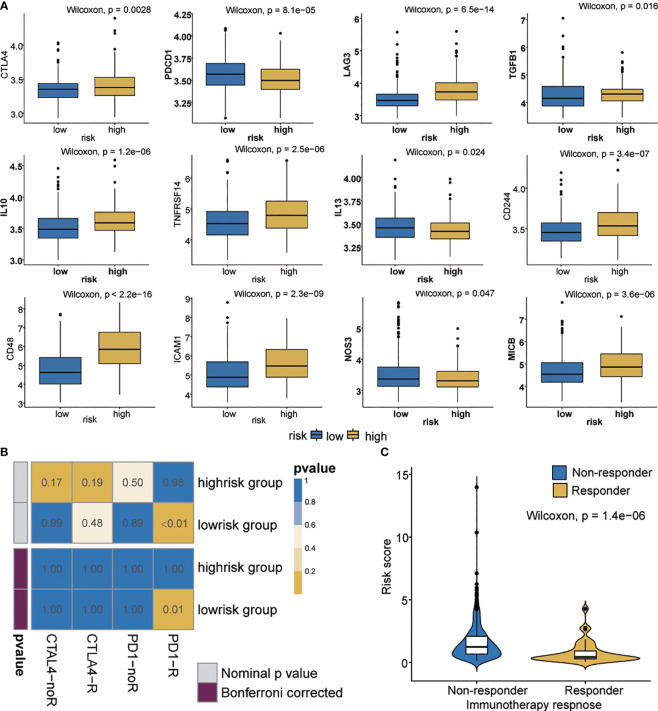
Immunotherapeutic responses in high- and low-risk groups with ovarian cancer. **(A)** Differential expression of 12 immune checkpoints. **(B)** Immunotherapeutic responses to anti-PD1 and anti-CTLA4 treatments. **(C)** Correlation between riskscore and immunotherapy response.

Immune checkpoint inhibitors using immunotherapies targeting PD-1, PD-L1, CTLA-4, and LAG3 have emerged as a promising strategy for the treatment of many diverse malignancies (49, 50). We evaluated the difference in expression between the high- and low-risk patients in 12 common immune checkpoints. The expression of PDCD1, IL13 and NOS3 in the low-risk group was significantly higher than that in the high-risk group (*p* < 0.05) (**Figure 6A**). We also evaluated the correlation between risk score and CTLA4 and PD-1 (**Supplementary Figures 6A, B**). Interestingly, CTLA4 in the high-risk group showed higher expression than that did the low-risk group (*p*<0.05). We also investigated the response to immune checkpoint blockade in high- and low-risk patients. We found that the low-risk group showed promising response to anti-PD-1 therapy (Bonferroni corrected *P* = 0.01, **Figure 6B**). Furthermore, patients’ responsiveness to immunotherapy was distributed in the low-risk but not in the high-risk group (**Figure 6C**). These results indicate that the risk score may predict response to immunotherapy.

### Establishment of a Risk Nomogram for Predicting Survival in Patients With OC

**Figure 7:**
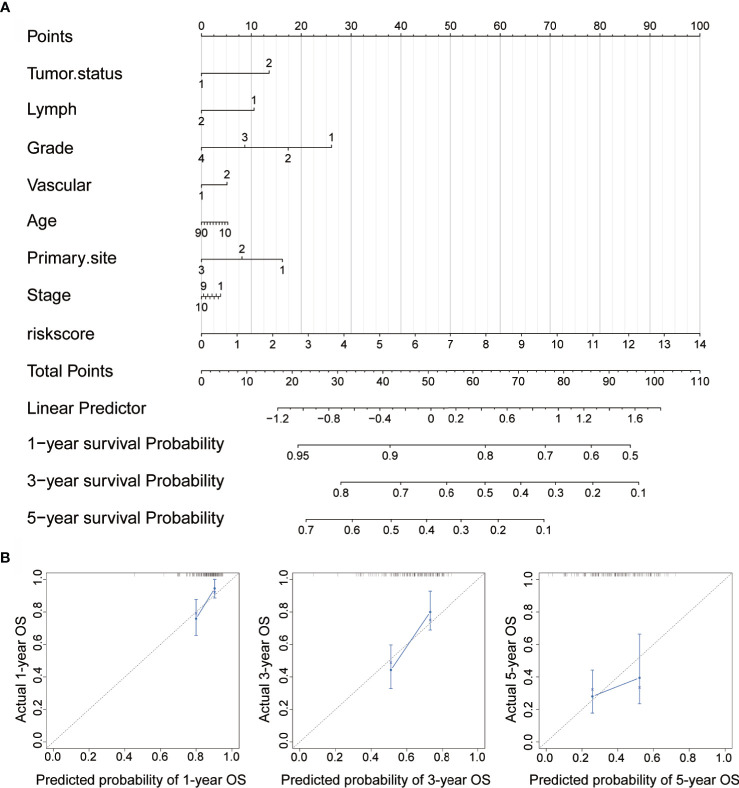
Construction of nomogram for survival prediction. **(A)** Nomogram combining the signatures with clinicopathological features. **(B)** Calibration plot showing that the nomogram-predicted survival probabilities correspond closely to the observed proportions.

To further enhance the predictive accuracy of the prognostic signature, we established a clinically adaptable nomogram by incorporating univariate clinicopathological features (e.g., tumor status, lymph node metastasis, grade, vascular invasion, age, primary site and stage). This provides clinicians with an effective tool to quantitatively predict the survival probability in patients with OC. As depicted in **Figure 7A**, by calculating the total score, we can estimate the probability of survival at 1, 3, and 5 years. A higher total score in the nomogram is related to poorer OS rates. Compared with other clinical factors, the risk score indicated higher accuracy.

To assess the discrimination and calibration abilities of the prognostic nomogram, calibration plots of the model for the 1-, 3-, and 5-year survival were constructed (**Figure 7B**). In the calibration analysis, the prediction lines of the 1-year, 3-year, and 5-year survival probabilities were very close to the ideal performance (45-degree dotted line), indicating that the accuracy of the nomogram is excellent. These results further strengthen the clinical significance of our proposed hypoxia-immune risk signature that exhibits an overall superior predictive power for determining survival outcomes in patients with OC.

## Discussion

Hypoxia induces and maintains malignant phenotypes, and is associated with poor clinical prognosis (51–53). At present, the tumor stage, grade, and lymph node metastasis have been recommended as independent prognostic factors for patients with OC. However, because of tumor heterogeneity, the screening for prognostic molecular markers that can fully reflect tumor biological characteristics is of great significance for improving individualized treatment strategies (54). Accumulating evidence suggests that hypoxia remains an important prognostic factor and an attractive therapeutic target.

Recently, a myriad of gene signatures has been identified to improve predictive prognosis in various types of tumors. For example, the 21-gene model and 18-gene model are used to provide breast (55) and colon cancer (56) recurrence scores, respectively. These results demonstrate that screening for new prognostic cancer markers based on gene expression profiles is a promising high-throughput molecular identification method, which is beneficial to clinical practice. Prognostic models based on hypoxia have been reported to have important clinical prognostic value in glioma and lung cancer (57, 58). Based on multi-omics data from the TCGA cohort, we conducted WGCNA to identify immune-related gene modules and found nine gene signatures using LASSO Cox analysis, a powerful dimensionality reduction method, with high AUC. In addition, risk scores and clinicopathological characteristics were used to construct a nomogram system that verifies the stability of the risk score and the accuracy of the survival prediction ability. With the availability of large data sets, the algorithm is effective and reasonable. Although further improvements are needed, our findings provide a theoretical basis for clinical applications.

Defects in antigen presentation, cytokine expression, and immune cell infiltration patterns show unique tumor escape mechanisms. Cytokines are important regulators of the immunosuppressive characteristics in a tumor microenvironment. Tumor-immunosuppressive cytokines can directly promote tumor cell growth, inhibit tumor cell apoptosis, and indirectly maintain an immunosuppressive microenvironment conducive to tumor growth by influencing angiogenesis and recruiting immune cells (59, 60). IL-10 is a key immunosuppressive cytokine secreted by M_2_ macrophages, Tregs, and Th2 cells and has been shown to impair the proliferation, cytokine production, and migration capabilities of effector T cells (61). Similarly, IL-6 and IL-32 participate in the immunosuppressive regulation of the tumor microenvironment. The activation of chemokines and their receptors, such as CCR7 and CCL21, are also associated with poor prognosis (62, 63). In OC xenograft, hypoxia promotes tolerance and angiogenesis *via* CCL28 and Tregs (64). Our findings showed that high-risk patients have increased levels of neutrophils, resting T and NK cells but decreased levels of activated cells. The immunosuppressive cytokines in the high-risk group were upregulated, which was consistent with the immunosuppressive function of this group.

SCNAs and somatic mutations at the genome level affect the efficacy of immunotherapy. The relatively low somatic point mutation frequency and high levels of SCNAs have been related to low immunogenicity in OC (65, 66). SCNAs are widely observed in OC, capable of indicating the gain or loss of chromosomes (67, 68). Focal-level SCNVs and arm-level SCNVs levels affect immune-escape markers. The immune microenvironment of tumors with high SCNAs levels is more tumorigenic and immunosuppressive than that with low SCNAs levels. Mutations or copy number changes drive immune cell infiltration (69). In both high- and low-level SCNAs tumors, the ratio between the mRNA levels of CD8^+^ T cell and Tregs genes was significantly reduced (70). In addition, SCNAs and somatic mutations affect tumor immunotherapy, and patients with high SCNAs levels respond poorly to immunotherapy (71). In our study, genomic analysis yielded distinct SCNAs and somatic mutation landscape, and identified significant differences in immune cell infiltration between the high- and low-risk groups that reacted differently to immune checkpoint blockers. Particularly, in the low-risk group, the expression of immune checkpoint PD-1 was significantly upregulated and responded well to anti-PD-1 inhibitors.

Increasingly, computational models are being used to evaluate the therapeutic efficacy of immunotherapy (72, 73).We used SubMap to predict the potential response to immunotherapy in OC patients. As expected, the patients in the low-risk group have a better response to anti-PD1 therapy than those in the high-risk group. Group with high TIDE score is more likely to induce immune evasion, indicating a lower response to immunotherapy (74), which is consistent with our findings.

The most attractive biomarkers for clinical applications are those that can provide patients with an accurate prognosis thereby assisting clinicians in choosing the most effective treatment. Our prognostic model consists of nine hypoxia-related genes, most of which have been studied as biomarkers for other cancer types, but rarely studied in OC. In lung cancer cells, *IGFBP2* induces erlotinib resistance by activating IGF-1R signaling (75). *SREBF1* and *SREBF2* are lipid metabolism regulators. Under oxygen-deprived conditions, the inhibition of *SREBP* prevents lipid biosynthesis in cancer cells and impairs cell survival (76). *TGFB1* signal transduction is related to adverse reactions to the PD-1/PD-L1 block (77). LAG-3 is a new generation of immune checkpoint that targets PD-1 and CTLA-4. Specifically, it targets the inhibitory receptors on the surface of T cells, and tumor cells evade immune surveillance by expressing LAG-3 ligands (78). Similarly, under hypoxic conditions, *PLK3* is a negative regulator of *HIF-1α*, and the expression of *HIF-1α* is closely related to the significant down-regulation of Plk3 expression in HeLa cells (79). *ANXA1* can regulate the activation and differentiation of T cells, promote their differentiation into Th1 cells, and negatively regulate their differentiation into Th2 cells (80). In colorectal cancer, the expression of *SLC1A1* is significantly and positively correlated with the level of CD8^+^ T cells and dendritic cell infiltration (81). Changes in the expression of *ALOX5AP* can lead to oxidative stress (82). In addition, in a recent biometric analysis, *ALOX5AP* was involved in the presentation of exogenous peptide antigens and antigen processing through MHC class II molecules (83). However, the biological functions of tumor hypoxia-related gene signatures need further exploration in OC.

In conclusion, this study identified the hypoxia genes of the modules most related to immunity, established a powerful hypoxia-related signatures for OC, explored the overall intensity of the immune response in the OC tumor microenvironment and escape mechanism, and predicted the responsiveness of immunotherapy. Our findings therefore not only offer reliable biomarkers for predicting the prognosis of OC, but also identify the responsiveness of patients to immunotherapy. Targeted hypoxia therapy for tumors may provide novel insights into individualized treatment strategies for OC patients.

## Data Availability Statement

The raw data supporting the conclusions of this article will be made available by the authors, without undue reservation.

## Ethics Statement

The transcriptome sequencing data and clinical data were collected from TCGA database. Written informed consent for participation was not required for this study in accordance with the national legislation and the institutional requirements.

## Author Contributions

Study concept and design: XC, HL, KC and SX. Analyzed, interpreted the data: XC and HL. Collection and assembly of data: XC, HL, DH, RX, YZ, YC, and HC. All authors contributed to the article and approved the submitted version.

## Funding

This work was supported by the National Natural Science Foundation of China (81874137), the science and technology innovation Program of Hunan Province (2020RC4011), the Outstanding Youth Foundation of Hunan Province (2018JJ1047), the Hunan Province Science and Technology Talent Promotion Project (2019TJ-Q10), Young Scholars of “Furong Scholar Program” in Hunan Province, and the Wisdom Accumulation and Talent Cultivation Project of the Third xiangya hosipital of Central South University (BJ202001), Independent exploration and innovation project for graduate students of Central South University (2021zzts1080), Philosophy and Social Science Foundation Project of Hunan Province (19YBA349), Clinical Medical Technology Innovation Guidance Plan of Hunan Province (2020SK53607).

## Conflict of Interest

The authors declare that the research was conducted in the absence of any commercial or financial relationships that could be construed as a potential conflict of interest.

## Supplementary Material

Supplementary Figure 1 Clustered dendrogram and Scatter plots of the highly correlated modules in different immune cells of OC. **(A)** Clustered dendrogram of 517 samples. **(B)** Red module has the highest association with activated mast cells. **(C–F)** Blue module is highly associated with T cells CD4 memory activated **(C)**, T cells gamma delta **(D)**, mast cells activated **(E)**, macrophages M1 **(F)**.

Supplementary Figure 2 Functional enrichment analysis. **(A, B)** GO and KEGG enrichment analysis results revealed that differential genes may be related to cytokine activity, hypoxia, PI3K-Akt, or other functional pathways.

Supplementary Figure 3 Correlations among 22 immune cells.

Supplementary Figure 4 Signal pathway enrichment analysis was performed on differential mRNAs in the low-risk and high-risk groups. **(A)** DEGs were reflected in volcano plot. The absolute value of log fold change > 0.5. **(B)** GO analysis results consisting of three parts: biological process, molecular function, and cellular component. **(C)** Partial display of the KEGG analysis results.

Supplementary Figure 5 Summary of mutation profiling in TCGA-OV samples from high- and low-risk groups. **(A)** Top 20 genes with the most significant mutations in high- and low-risk groups. The bar chart shows the total number of mutations in the top 20 genes of each patient. The bar chart on the right shows the number of samples in which 20 genes were mutated in all samples. The different colors in the thermogram indicate the type of mutation; gray indicates no mutation. **(B)** Statistical analysis of mutation types based on different categories, wherein missense mutation is the most frequent; SNP has more frequency than deletion or insertion; and C > T is the most common type of SNV. **(C)** Box plot summarizing the SNV of TCGA-OV cohort. Box plot created by dividing the SNV into transition (Ti) and transversion (Tv). Bundled column chart showing the SNV classification of individual cases.

Supplementary Figure 6 The relationship between CTLA4 and PD-1. **(A)** The relationship between risk score and CTLA4. **(B)** The relationship between risk score and PD-1.

Supplementary Table 1 Amplification or deletion genes in high- and low-risk groups.

## Abbreviations

OC, Ovarian cancer; OS, Overall survival; TCGA, The cancer genome atlas; WGCNA, Weighted gene co-expression network analysis; LASSO, Least absolute shrinkage and selection operator; TIMER, Tumor Immune Estimation Resource; TIDE, Tumor immune dysfunction and exclusion; GO, gene ontology; KEGG, Kyoto Encyclopedia of Genes and Genomes; GSEA, Gene set enrichment analysis; ROC, Receiver operating characteristic; TIM, Tumor immune microenvironment; TME, Tumor microenvironment; CTLA-4, Cytotoxic lymphocyte antigen-4; PD-1, Programmed cell death protein 1; PD-L1, Programmed-death ligand 1; LAG3, Lymphocyte Activating 3; MHCI, Major histocompatibility complex class I; MHCII, Major histocompatibility complex class II; APCs, Activate antigen presenting cells; DEGs, Differentially expressed genes; NES, Normalized Enrichment Score; TMB, tumor mutational burden; SCNAs, somatic copy number alterations.
