# Supplementary figures and images for: Multi-Omics Profiling Identifies Risk Hypoxia-Related Signatures for Ovarian Cancer Prognosis
Source: Front Immunol. 2021 Jul 19;12:645839. doi: 10.3389/fimmu.2021.645839 (PMC8327177; doi:10.3389/fimmu.2021.645839)

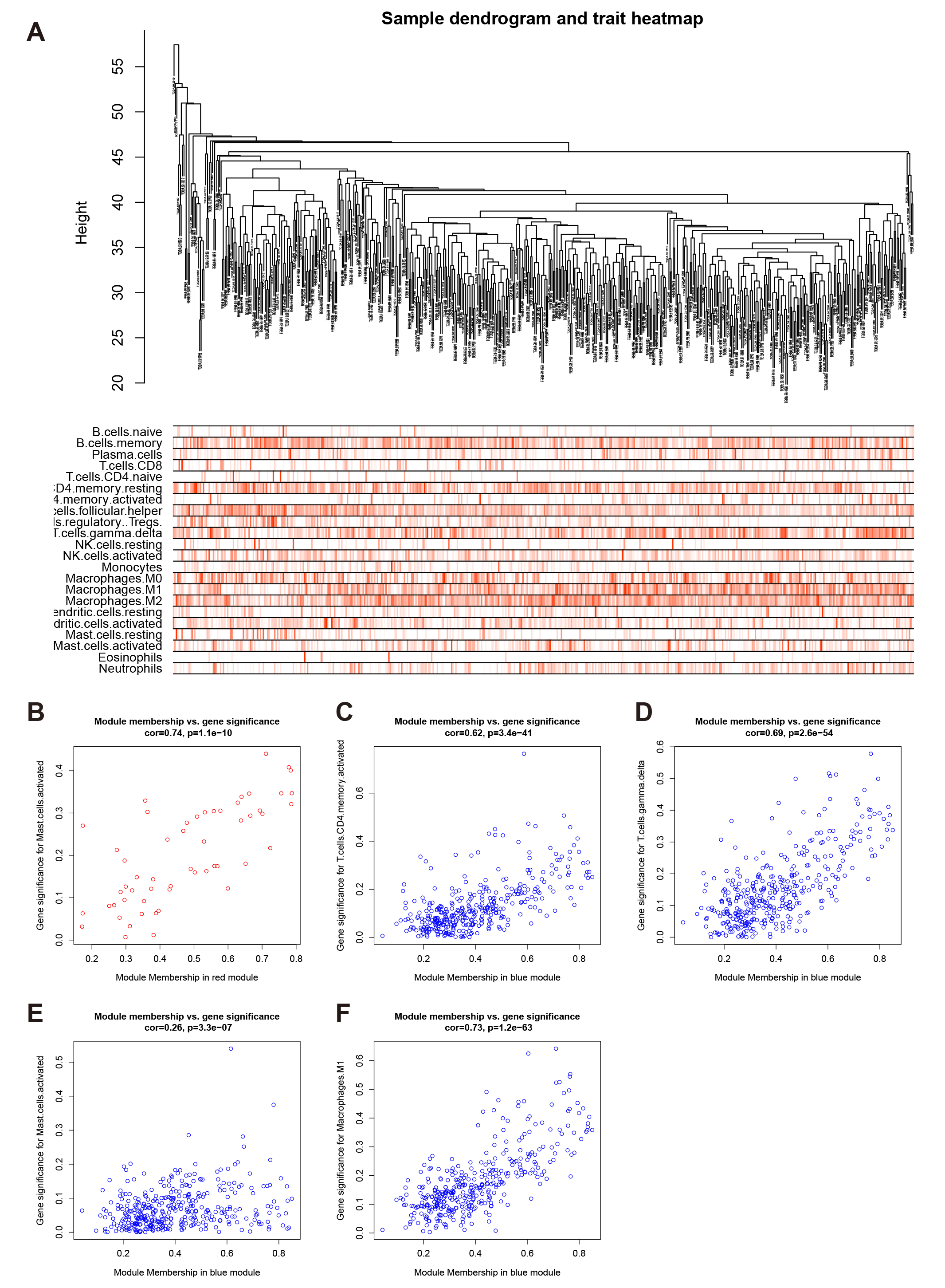

Supplement: Supplementary Figure 1 — Clustered dendrogram and Scatter plots of the highly correlated modules in different immune cells of OC. (A) Clustered dendrogram of 517 samples. (B) Red module has the highest association with activated mast cells. (C–F) Blue module is highly associated with T cells CD4 memory activated (C), T cells gamma delta (D), mast cells activated (E), macrophages M1 (F). [file Image_1.tif]

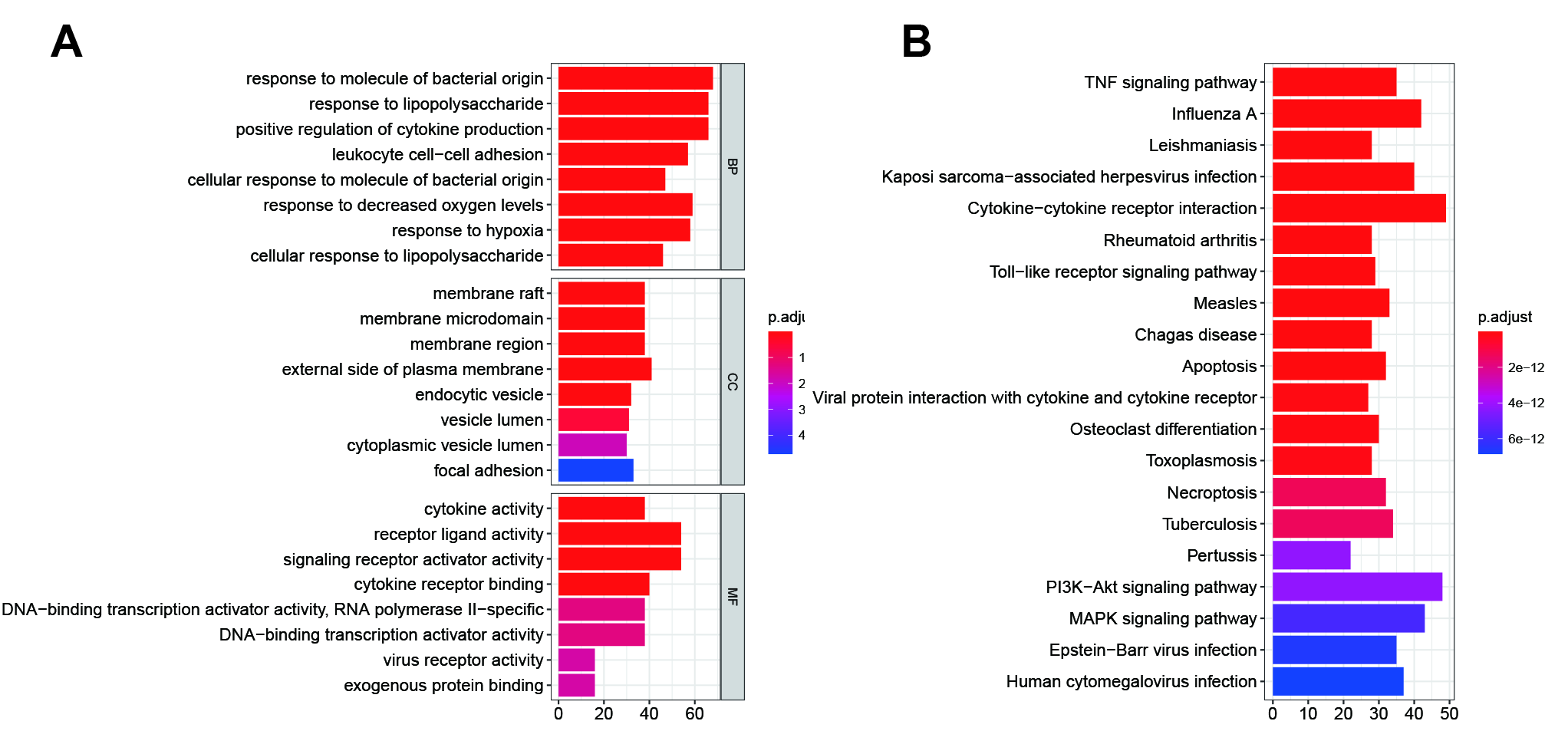

Supplement: Supplementary Figure 2 — Functional enrichment analysis. (A, B) GO and KEGG enrichment analysis results revealed that differential genes may be related to cytokine activity, hypoxia, PI3K-Akt, or other functional pathways. [file Image_2.tif]

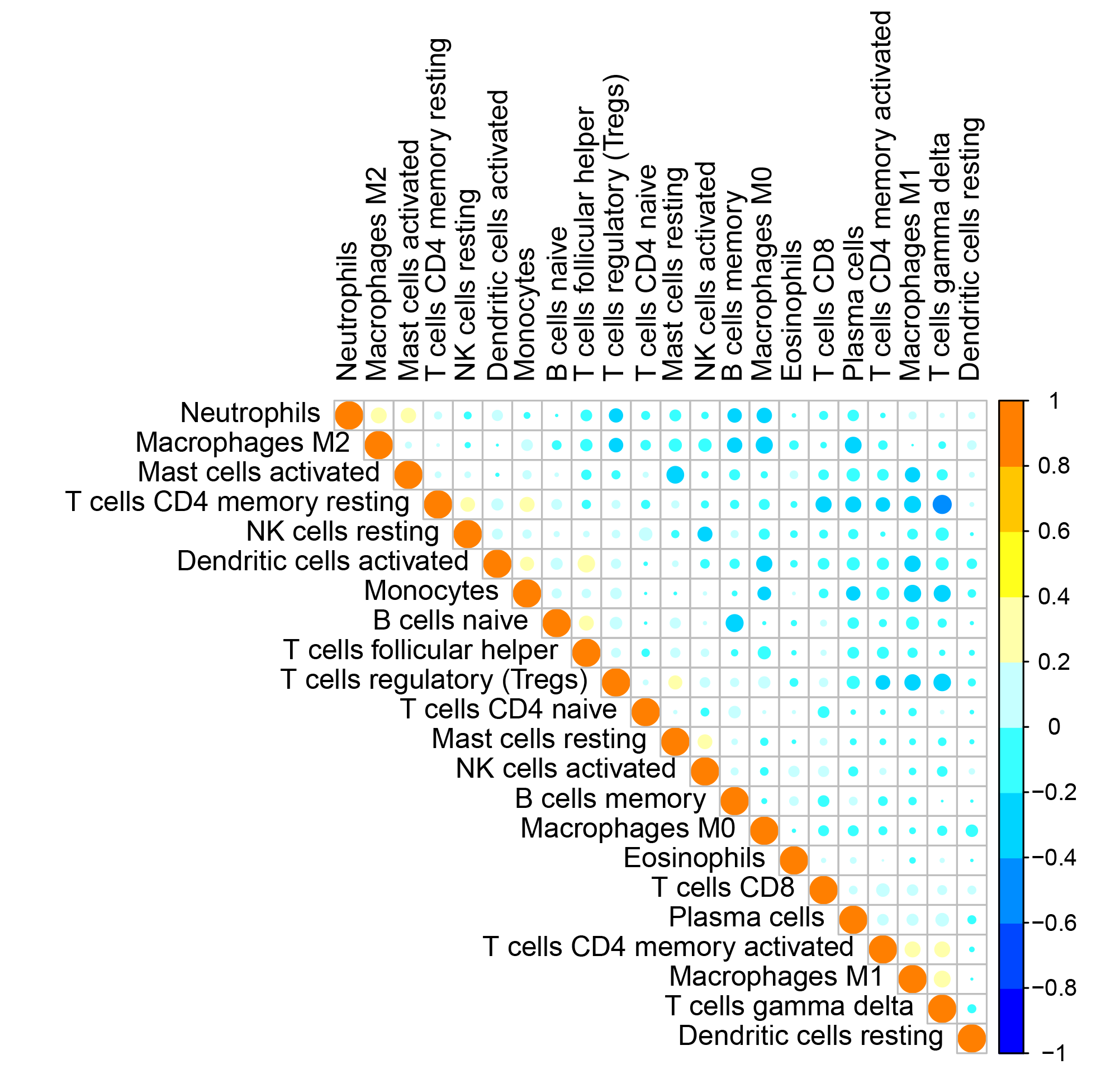

Supplement: Supplementary Figure 3 — Correlations among 22 immune cells. [file Image_3.tif]

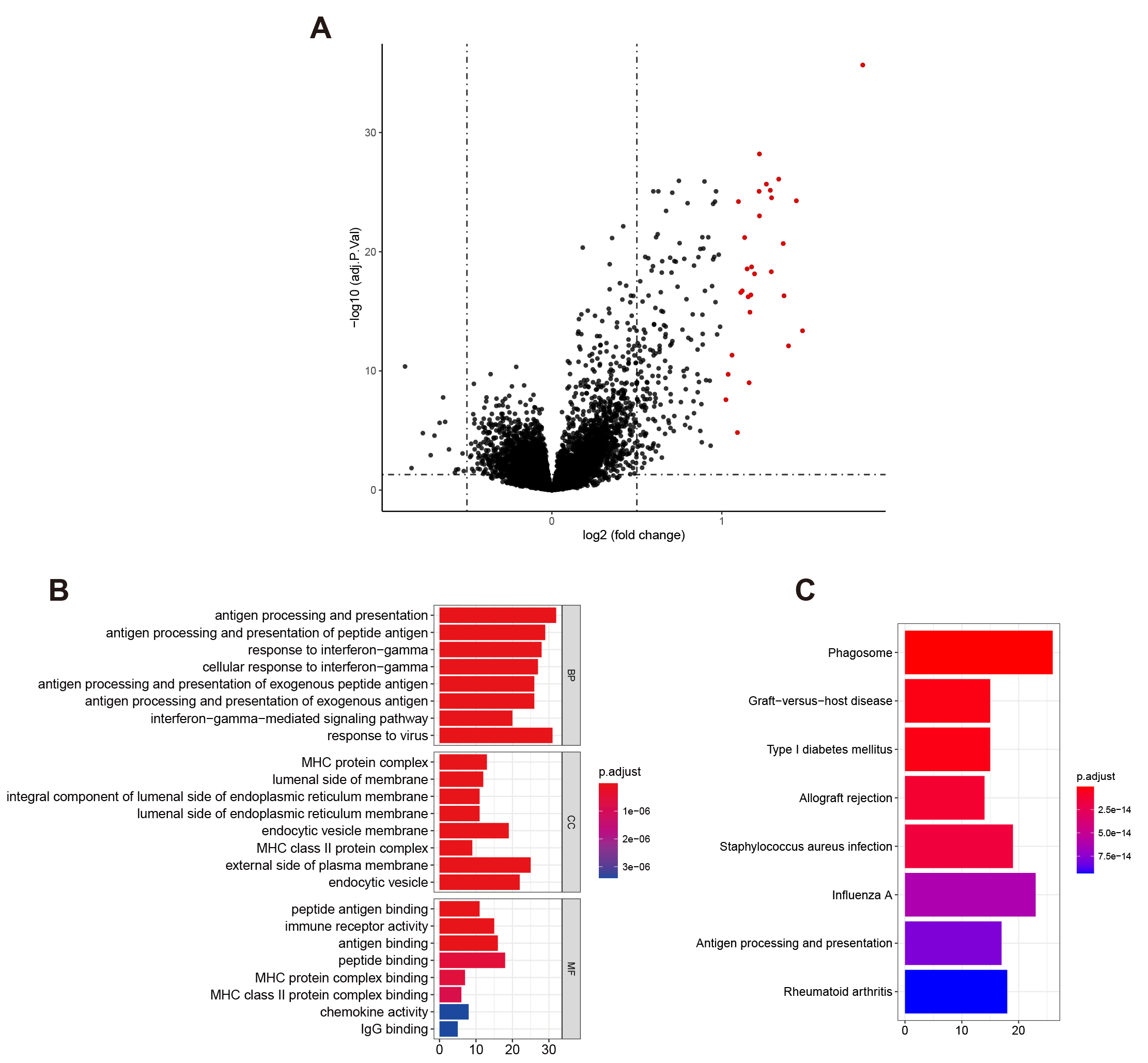

Supplement: Supplementary Figure 4 — Signal pathway enrichment analysis was performed on differential mRNAs in the low-risk and high-risk groups. (A) DEGs were reflected in volcano plot. The absolute value of log fold change > 0.5. (B) GO analysis results consisting of three parts: biological process, molecular function, and cellular component. (C) Partial display of the KEGG analysis results. [file Image_4.tif]

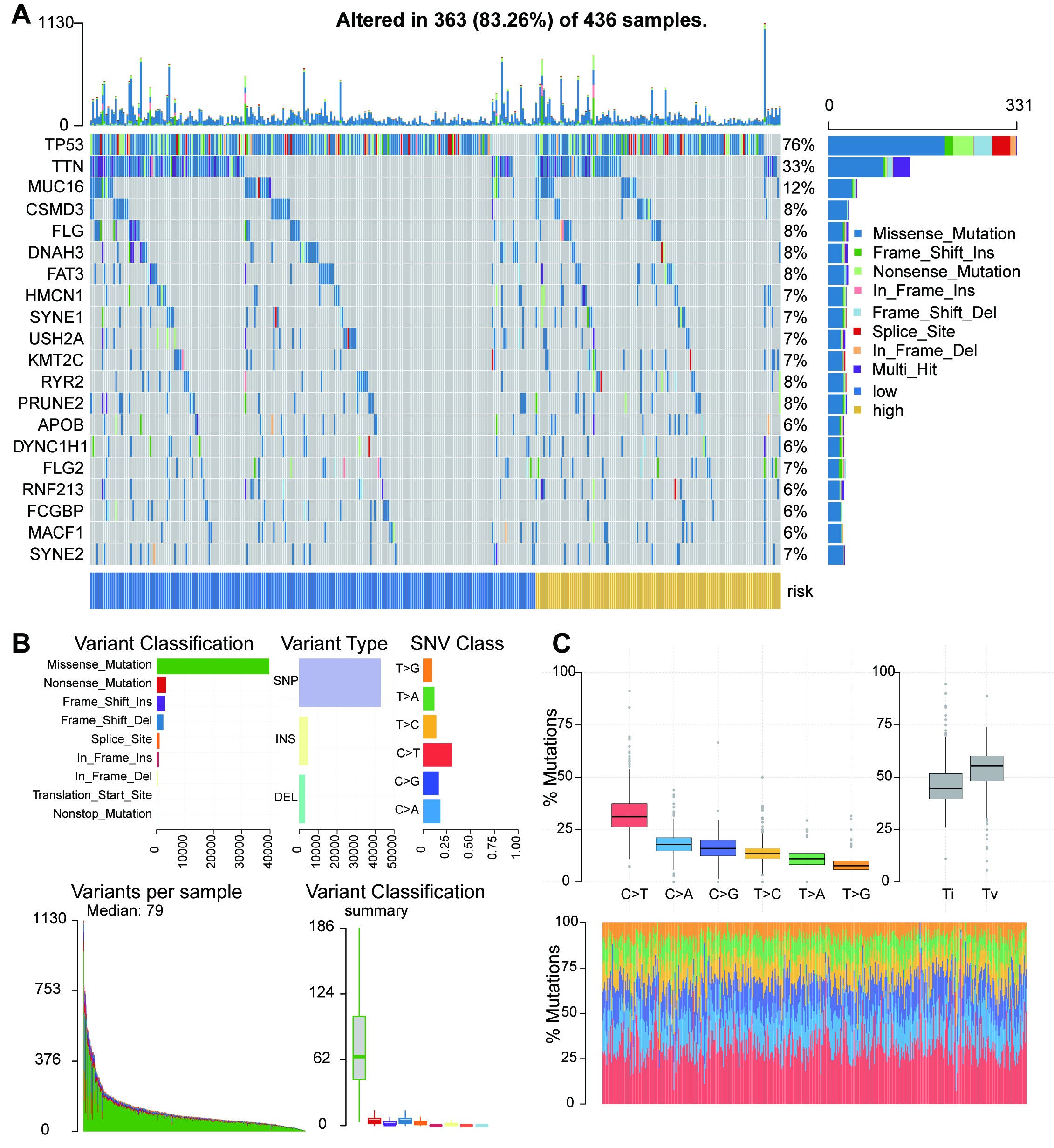

Supplement: Supplementary Figure 5 — Summary of mutation profiling in TCGA-OV samples from high- and low-risk groups. (A) Top 20 genes with the most significant mutations in high- and low-risk groups. The bar chart shows the total number of mutations in the top 20 genes of each patient. The bar chart on the right shows the number of samples in which 20 genes were mutated in all samples. The different colors in the thermogram indicate the type of mutation; gray indicates no mutation. (B) Statistical analysis of mutation types based on different categories, wherein missense mutation is the most frequent; SNP has more frequency than deletion or insertion; and C > T is the most common type of SNV. (C) Box plot summarizing the SNV of TCGA-OV cohort. Box plot created by dividing the SNV into transition (Ti) and transversion (Tv). Bundled column chart showing the SNV classification of individual cases. [file Image_5.tif]

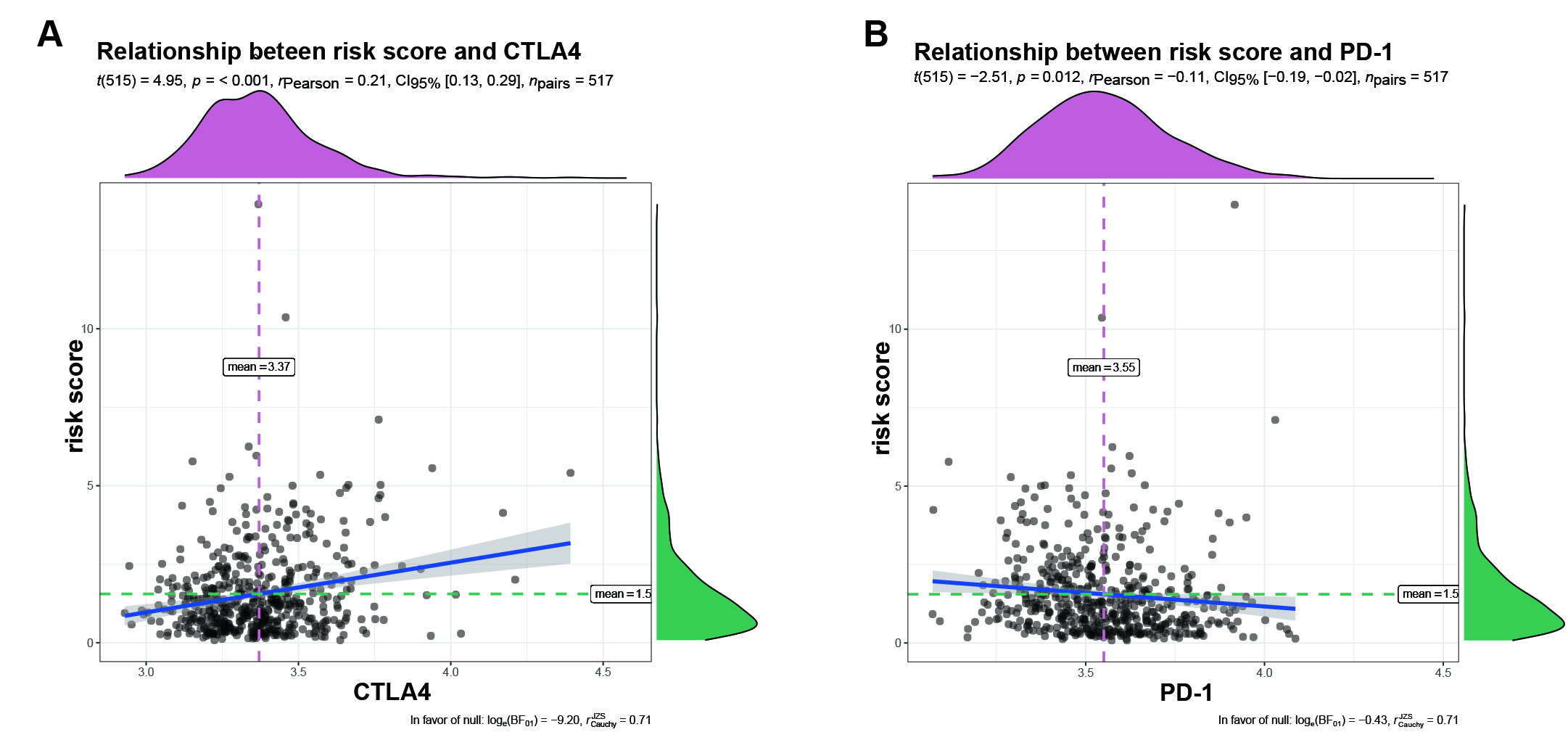

Supplement: Supplementary Figure 6 — The relationship between CTLA4 and PD-1. (A) The relationship between risk score and CTLA4. (B) The relationship between risk score and PD-1. [file Image_6.tif]
